# Political motivation as a key driver for universal health coverage

**DOI:** 10.3389/fpubh.2022.922578

**Published:** 2022-11-15

**Authors:** Sandhya Venkateswaran, Shruti Slaria, Sampriti Mukherjee

**Affiliations:** ^1^Centre for Social and Economic Progress, New Delhi, India; ^2^Lancet Citizen's Commission on Reimagining India's Health System, New Delhi, India; ^3^Swaniti Initiative, New Delhi, India

**Keywords:** political economy, health prioritization, India, political motivation, health reforms

## Abstract

Variation in public investments to health, health outcomes, and progress toward universal health coverage across countries is vast and neither economic status nor the knowledge on solutions have borne out to be binding constraints to health improvements. The drivers for universal health coverage go beyond the macro-economic context of a nation, and as pointed out by scholars, are deeply linked with the extent of political prioritization of healthcare. Low public investments to health in India and slow movement toward universal health coverage underline the need for more attention to the political priority accorded to health in the country. While the role of politics in policy reforms has been established by several scholars, this paper seeks to identify the intrinsic motivations or incentives that drive political priority. Drawing on the experience of nine countries, the paper attempts to inform the analysis for countries such as India (where progress toward universal health coverage remains slow), on the political incentives for prioritization of healthcare, and how these may be shaped or strengthened. The analysis finds that health care reforms happen in (at least) two stages: the existence and recognition of a national context and a problem, followed by political opportunities and motivations which lead political leaders to address the identified problem. The paper separates motivation as a distinct factor for analysis because, in the absence of strong incentives, not every political opportunity may lead to attention to an issue, and finds that reforms were motivated by a need to gain political legitimacy by an incoming regime, or by its political ideology, or a combination of both. Importantly, political motivation does not always take root in itself, but often driven by external factors and stakeholders who contribute to creating or strengthening incentives for political attention. A greater role from citizens and other actors such as elected representatives, questioning status quo and highlighting the schisms in the social contract between a political regime and citizens may contribute to shifting the source of legitimacy for leaders.

## Introduction and research question

The world has made significant strides in health, in eliminating disease and improving health indicators and in committing itself to the Sustainable Development Goals, with several countries aspiring to universal health coverage. However, approximately half of the world's population is still unable to obtain essential health care services, and every year about 100 million people are pushed into extreme poverty globally, because of healthcare-related expenditures (1).

Variation in public investments to health, health outcomes, and progress toward universal health coverage across countries is vast and neither economic status nor the lack of understanding on solutions have borne out to be binding constraints to health improvements. With sharing of global knowledge and expertise, it is not the lack of understanding that is the binding constraint to health improvements at a country level. With progress in low and lower-middle-income countries such as Indonesia, Vietnam, Brazil, Turkey, Thailand it is also not the economic status that is holding back health improvements. Mor (2) notes that total health expenditure (as a proxy for economic status) alone explains only about 50 percent of DALY rates. Several countries across the world, facing challenges of less than strong economies and high inequality in access to healthcare, have successfully managed to prioritize healthcare and move toward universal health coverage. Clearly, the drivers for universal health coverage go beyond the macro-economic context of a nation and the availability of solutions. A key driver, as has been pointed out by several scholars, is the extent of political attention and prioritization in influencing progress on health (3–5). Attention by political leaders and policy makers increases the probability of policy reforms and public investments needed for progress on health (reforms that may otherwise have been deprioritised in a policy space crowded with many competing issues). Public investments to health have remined low in India and movement toward universal health coverage slow; pointing to the need for more attention to the political priority accorded to health in the country.

While the role of politics in policy change has been established by several scholars (6, 7) this paper seeks to identify the motivations that drive political attention and prioritization by country leaders. Through the experience of several countries, it attempts to inform the analysis for countries such as India and others (where progress toward universal health coverage remains slow), of the political incentives for prioritization of healthcare, and how these may be shaped or strengthened.

There is a vast body of literature on solutions to many of the healthcare challenges across countries, and the nature of reforms undertaken. This paper does not focus on those. Because scholarship on the political motivations for reform and for health prioritization is limited, especially in a context of competing national priorities, that constitutes the focus of this paper.

With considerable scholarship on how attention of political and other leaders drives policy reform and public investments, a deeper question points to examining the forces that lead to such attention. Much has been written about external drivers, but we hypothesize that there are intrinsic motivations and incentives that draw the attention and commitment of political leaders to an issue, in this case health. The focus of this analysis is to identify the motivation of country leaders and policymakers in prioritizing health, and the factors that contribute to it.

This analysis should not be interpreted to suggest that the entire process of health priority and health sector reform is entirely driven by the intrinsic motivation of country leaders. Building on political economy frameworks developed by numerous scholars, policy entrepreneurs have contributed in various forms to facilitate and promote the processes of reform initiation. But it can be argued that all such efforts are successful when there is a clear incentive for country leaders, who need to weigh choices and priority across multiple national competing demands and needs. It is precisely the understanding of such motivation or incentives that is the focus of this paper, and the external factors, stakeholders and processes that play a role a role in creating these incentives.

## Research question and methodology

We use political attention and political priority for health as interchangeable. Building on a definition by Shiffman and Smith (3) referred to in Schmidt et al. (8), we view political priority as the degree to which (1) political leaders actively pay attention to health and prioritize interventions needed for progress on health, (2) political decisions lead to system reforms and programs that address the problem, and (3) reforms and programs are supported by financial and other resources.

This analysis of the incentives for health prioritization was done across nine countries—Turkey, Mexico, Brazil, Argentina, Indonesia, Philippines, Thailand, Vietnam, and China. Two sets of criteria influenced the selection of these countries. One, they had undertaken health reforms, with country leadership demonstrating a priority for health. Two, countries were selected to represent different economic status, political systems and geographic regions.

As per the first criteria, the countries were selected because they demonstrated political priority to health during a specific time period, which in this case was determined by the factors mentioned above, (1) political leaders actively pay attention to health and prioritize interventions needed for progress on health, and (2) political decisions lead to system reforms and programs that address the problem. All of the countries selected had undertaken health reforms and initiated programs to address health related challenges at a specific time.

- Turkey faced inequities in health outcomes across regions and segments of the population, shortage and inequitable distribution of infrastructure and human resources, and inequitable financing of the health system. These were addressed through the Health Transformation Plan which introduced a single purchaser model to address these inequities;
- Thailand had an uninsured population of 30 percent, with significant private expenditure on health, leading to the introduction of a tax financed program (the 30 Baht scheme) providing health care at the point of service for a co-payment of 30 Bahts;
- In Argentina, the economic crises led unemployment resulted in a large proportion of the population losing health insurance cover, and consequently deteriorating health outcomes. This was addressed through Plan Nacer, aimed at increasing coverage of basic services among the uninsured population;
- A large proportion of Mexico's population, specially the poorest, lacked health insurance, leading to high out of pocket expenditures and catastrophic financial events. Reforms in the form of Seguro Popular were introduced to address the unequal distribution of the financial, physical and human resources in health services;
- Brazil had different health rights for workers and poor populations working outside the formal economy, which was addressed through a Unified Health System enshrined in the constitution to ensure equal access to health services for all citizens;
- In Vietnam, out of pocket expenditure on health increased significantly with a shift to a market economy. A state financed health insurance was introduced along with citizens being provided a legal right to health protection;
- The Philippines healthcare program was not successful in addressing the healthcare needs of its poor due to poor governance and accountability. The PhilHealth sponsored program was extended to focus on the poorest population, with premiums paid for by the government;
- Indonesia experienced an increased cost of healthcare input and diminishing purchasing power after the financial crisis. A constitutional amendment made the state responsible for ensuring health service provision for all citizens, leading to the national government paying for inpatient services for all poor people;
- China witnessed a shift to a market based system, resulting in inequalities in health access and increased private health expenditure. A basic health insurance scheme was introduced in response.

Second, the country selection was aimed at obtaining a representative sample of diverse economic, political and geographic contexts. On the economic front, countries were chosen from amongst low income, lower middle income, and upper middle income to gauge the interaction of the economic circumstance with the motivation to reform domestic healthcare sectors. The rationale for different economic contexts stems from the need to explore hypotheses pointing to stronger economic contexts being more amenable to the introduction of reforms. The countries chosen represent significantly different economic contexts with per capita GDP ranging from $430.05 to $7,484.49 at the time of initiating health reforms (9). At the time of the countries' healthcare reforms (as detailed in later sections), the World Bank classified Vietnam and Indonesia as low income countries; Philippines, China, Brazil, Turkey, and Thailand as lower middle income countries; and Argentina and Mexico as upper middle income countries (9).

The dynamics of engagement between political leaders and citizens could vary according to the type of political regime which drives principal agent relationships and political incentives. It is for this reason that political context could be viewed as another variable for issue prioritization, where different political regimes, democratic and authoritarian, may respond to citizen needs differently. This could then suggest very different factors leading to political attention to an issue across political systems. On the political front therefore, countries were selected to represent varied political systems (democratic regimes, single party led countries, and those moving toward democratization) to examine if and how the political system influences the motivation for and priority to health issues.

Geographic regions have experienced economic and/or political transitions: structural shifts emerging from the Washington Consensus, the transition from authoritarian rule in much of Latin America and the Asian financial crisis in South-East Asia, to name a few. These processes shaped the autonomy and priorities of countries in the region. This analysis therefore includes countries across regions, in Latin America, Middle East, and Asia, to examine how the regional contexts influenced the rationale and motivation for attention to health.

The study has followed a mixed methodology of extensive secondary literature analysis and a limited set of stakeholder interviews. The secondary analysis focused on specific reforms introduced in the country in the last few decades (recognizing that several countries have undertaken multiple reforms across years). It examined contexts pre and post reforms, and combined historical, political, economic and social aspects to trace the trajectory of the processes that led to reforms. Stakeholders interviewed^1^ included a former bureaucrats, researchers and officials from multilateral organizations who were engaged with these countries in varied ways. The analysis is limited to the study of the priority given to health by political and other leaders and the resulting initiation of sector reforms, rather than their actual implementation.

## Results

**Figure 1:**
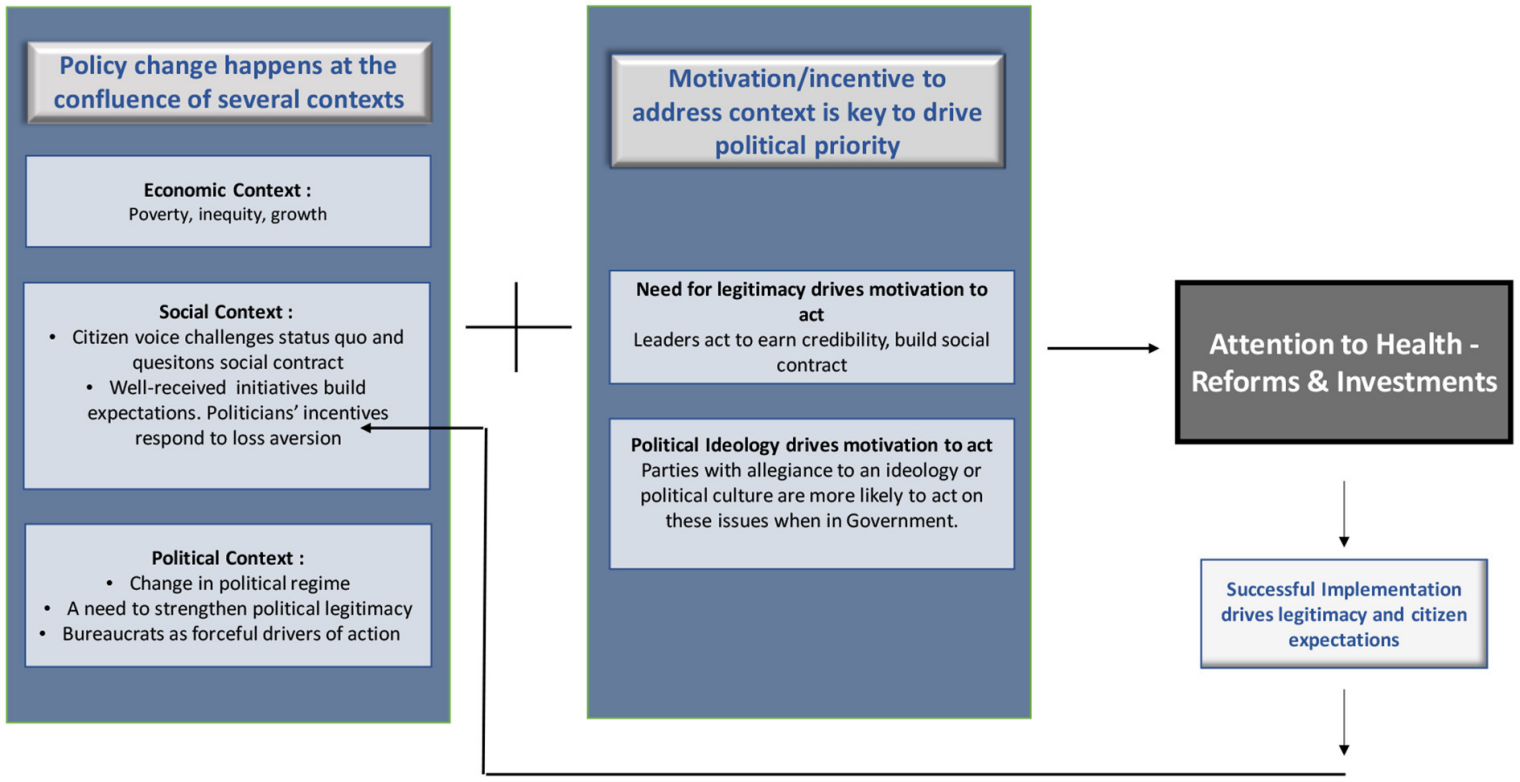
Framework for political attention to health.

This paper rests on the basic premise that health care reforms happen in (at least) two stages. Figure 1 provides a framing of this. The first is the existence and recognition of a national context and a problem, which in most countries analyzed was high poverty and/or inequality, of which a tangible component is unequal access to quality healthcare services. The economic context, in terms of the extent of poverty and inequality, determines not merely income levels, but access to health services, education opportunities, housing and multiple other factors that constitute determinants of social equity and quality and dignity of life. The fulfillment of universal health coverage therefore is intricately linked with the economic context, which may drive both, access to health services and also the country's ability to provide universal coverage.

This is followed by political opportunities and motivations which lead policy makers and political leaders to address the national context/problem. This paper separates motivation as a distinct factor for analysis because not every political opportunity may lead to sectoral attention, in the absence of strong incentives. Elections, as a political window, underlines this fact, as not every change in political leadership leads to a shift in a sector's priority.

The experience across the nine countries analyzed also underlines the influence of other factors and stakeholders, such as civil society organizations, social workers, and activists; social movements; citizen demand; and international organizations, in influencing agenda setting; a role identified by many political economy scholars (10–14). Much of the scholarship has demonstrated ways in which a country's social context and processes hold the potential to determine and drive the social contract between citizens and political leaders, which influences the issues that get prioritized at a political level.

The study sought to understand the factors that drive political prioritization of policy reforms, with an emphasis on healthcare. The paper explores motivations to address specific economic and socio-political contexts, and the nature of incentives that move leaders from recognizing a situation to taking action on it. Despite similarities in economic contexts, we examined if, and the manner in which, incentives varied for different leaders.

The goals of reducing poverty and inequality or seeking national development or increased growth are seen across several countries. The pathways to these, however, vary and are often built around the perceived incentives by leaders. Incentives could be viewed in different forms; it is precisely this interrogation that led to identifying the foundational motivation for leaders.

Buchanan (15) points to actions of leaders that are aimed at “morally” justifying their governance and in turn leading citizens to provide validity to the government's administrative decisions. This is seen in various political regimes; to appease the electorate in democratic systems and to validate the performance of autocratic systems.

A different pathway is based on ideology, as defined by several political theorists (16–18), built on socially shared philosophies of life which set about ideals about structuring the proper order of society and processes to achieve the same.

In order to understand how these and possibly other pathways influenced the political prioritization of health in these countries, it is important to understand the national and political context in each.

### Economic context

At the time of reforms, all nine countries were experiencing high rates of poverty or inequality or both, along with high levels of out-of-pocket expenditures on health. In large part, healthcare reforms were introduced by them to address systemic inequities constraining Universal Health Coverage.

At the time reforms were undertaken, Turkey had a Gini coefficient at 0.43, with only 66.3% of the population covered by insurance. The 1990's were characterized by a series of weak and indecisive coalition governments, resulting in unstable and unsustainable economic development, with the country witnessing economic cycles of boom and bust. As a result, Turkey witnessed a contraction in its real GDP during this period, rampant inflation and high rates of unemployment. Inequality increased in the country with the Gini coefficient rising to 0.43 in the early 2000's. Marred by political instability, economic shocks, runaway inflation, rising unemployment, and social discord, successive governments in the 1990's failed to prioritize the health sector, which faced three major issues - inadequate and inequitable financing of the health system, absolute shortage and inequitable distribution of physical infrastructure and health human resources, and inequities in health outcomes, between the deprived eastern areas and the more developed western regions of the country, among the richer and poorer segments of the population, and across rural and urban areas (19).

Mexico had a Gini coefficient of 0.50 with out-of-pocket expenditure at 54% before the reforms. The Mexican health system was designed to provide episodic and acute care. However, declining fertility rate and increasing life expectancy brought about an epidemiological transition in the country, increasing the burden of noncommunicable disease and chronic illness which the health system was ill-equipped to deal with (20). By the mid-1990's, approximately half of Mexico's population lacked health insurance, including about 2.5 million families from the poorest sections of society, who had access only to very basic community and preventive health interventions included in the poverty alleviation programme (21). Consequently, more than half of the total national health expenditure was out-of-pocket. These high levels of OOP were exposing Mexican households to catastrophic financial events, and in 2000, approximately 3 to 4 million Mexican families (approximately 4% of the total population) incurred impoverishing health expenditures (22). Several financial imbalances prevented the health-care system from responding to population health including, (1) the low level of overall health spending; (2) imbalance in allocation of public resources between the insured and uninsured, and among states; (3) inequitable contribution of states to finance health care—with significant differences in expenditure per head across states; and (4) chronic under investments in health infrastructure (21, 23).

Thailand had a Gini coefficient of 0.42 and 34% of health spend was paid out of pocket when the 30-baht reforms were introduced. While Thailand had always focussed on providing healthcare to its citizens, including the launch of the Low-Income Scheme in 1975, 30% of the country's population remained uninsured by 2001. Kuhonta (24) argues that the introduction of the 1997 constitution and the Asian financial crisis were the broad macro factors that created the conditions for the implementation of UHC in Thailand. While the Asian Financial Crisis underlined the need for social equity, the constitution created conditions for government stability and hence for policy sustainability.

The Philippines saw improvement in economic growth since 2001, but this did not translate into inclusive development and reduction of poverty. The country had a Gini Coefficient of 0.46 with out-of-pocket health expenditures reaching 55.57% in 2009 (a year before the reforms were initiated). Inequalities persisted, of which one was access to quality healthcare, reflected through high out-of-pocket health expenditures, one of the major factors that led to the impoverishment of poor households (25, 26). The Gloria Macapagal Arroyo administration (2001–2010) did little to address this, and against whom several allegations of corruption and human rights abuses had been leveled.

Brazil had a Gini coefficient of 0.61 when it underwent the democratization process in 1988 (9). The country underwent industrialization and urbanization from the 1930's to 1980's. This led to a change in demographic patterns due to increase in incomes, low fertility, declining mortality, and increasing life expectancy. Consequently, Brazil witnessed an epidemiological transition marked by the rise in cardiovascular illnesses, cancer diseases, and other non—communicable diseases (27). At the same time, the 20 years of military rule from 1964 to 1985 were characterized by an increased focus on economic development as opposed to social welfare. The private sector grew, including the private provision of publicly financed care through social security arrangements, and those working in the informal sector and urban and rural poor were largely excluded from the same. Consequently, even public sector healthcare was concentrated in the developed parts of the country and excluded the urban and rural poor (28). When the country prepared for a transition from an authoritarian to a democratic regime after 20 years of military rule, “health sector reform became a fundamental feature of the fight to re-democratize the society and the political regime” (28).

In Indonesia democratization was ushered in after the Asian Financial Crisis, when the percentage of people living under the $1.90 poverty line (2011 PPP) was as high as 63.2% with out-of-pocket health expenditures comprising 43.86% of total health expenditure. The Asian Financial Crisis of 1997, citizen's protests and political instability acted as a catalyst for the reform in Indonesia's healthcare system. Prior to the crisis (1970–1996), Indonesia's health outcomes were relatively better than other peer countries (29). Post the economic crisis, two events impacted the poor in a big way. Indonesia faced devaluation of currency and inflation, leading to an increase in the prices of health care inputs, especially those of imported pharmaceutical products. Reduced tax revenues led to reduced health expenditure by the government, in turn leading to shortage of medicines and equipment in government health facilities. This impacted usage of government run facilities, leading to the worsening of the health status of the population (30). Second, the crisis pushed an additional 36 million Indonesian people into absolute poverty. This led to an adverse impact on poor households who had to simultaneously contend with diminishing purchasing power as well as increased cost of treatment at Indonesian government health centers (government facilities charged user fees from patients).

Like in other countries in the region, Argentina's health sector agenda was developed in the 1990s at the same time as the country was experiencing “profound economic and social restructuring, along neoliberal lines” (12), and it is important to understand health sector reforms in Argentina vis-à-vis the wider context of neo-liberal restructuring and governance. Between 1989 and 1999, in collaboration with the World Bank and IMF, the Carlos Menem administration adopted neoliberal reforms that involved trade liberalization and privatization, also reflected in the country's healthcare reforms (31). The reforms however failed to bring about substantial results and the hyperinflationary economic crisis from 1999 to 2002 led to a public health emergency. The GDP of the country fell by 18.3 percent between 1998 and 2002; the number of poor grew by 20 percentage points and inequity worsened. As unemployment increased and more people were laid off from their jobs, approximately 12 percent of the workers lost their health insurance cover and the sharp fall in employment rates resulted in 60 percent of the total population outside the social health insurance system (32). The crisis resulted in deteriorating health indicators, including child and maternal mortality rates especially in the poorest regions.

Vietnam, being a Communist one-party led state, had an inherent mandate of providing access to healthcare to all its citizens as part of its socialist agenda. Despite this, 37% of the population lived under the $1.90 poverty line in 2002, a year before the reforms were carried out (2011 PPP) (9). In late 1980s, Vietnam was hit by a socio-economic crisis after the collapse of the Soviet Union which reduced foreign aid. This affected the government's ability to solely fund health care activities and ushered in a market economy policy with a socialist government structure (33). This led to high out of pocket expenses on healthcare, at 37.14 percent of total healthcare expenditure, as of 2002 (9).

In China, out-of-pocket expenditure was at 64.19% of total health expenditure while 31.7% of the population lived under the $1.90 poverty line, as of 2002, just before the health reforms were implemented (2011 PPP) (9). The health privatization policies of a market-led system (discussed later in this paper) followed by the Deng Xiaoping administration led to a reduction in government regulations within the healthcare sector and re-orientation of public hospitals into for-profit entities. These shifts led to health inequalities between rural and urban residents, poor quality of healthcare and increasing private health expenditure (34).

### Political context

As with the economic context, similarities in political context across the nine countries were evident from the political transitions they went through, although the nature and extent of political change was quite different. Some countries witnessed the initiation of a democratic process (such as Brazil and Indonesia), while others merely witnessed a change of political leadership (Turkey, Thailand, Mexico, Argentina, China, Philippines).

#### Democratization

In Indonesia, the aftermath of the economic crisis led to widespread social unrest and citizen's protests. This unrest was instrumental in forcing the then autocratic ruler, President Suharto, to step down in 1998. While his successor, B.J. Habibie, tried to mitigate the effects of the crisis and increase acceptability to the ruling party by strengthening education, nutrition, and health services for the poorest, these did not prove effective in saving Suharto's party (29, 35–37).

In order to counter Suharto's autocratic policies, multiple student movements had been unified into a political party known as the Indonesian Democratic Party of Struggle (PDI-P party); a party formed by Megawati Sukarnoputri (daughter of former President Suharto) as a government dissent faction in 1998. Although this party did not have an inherent welfarist agenda, they capitalized on citizens' protests against President Suharto. As demands for democratization increased, they highlighted the social ills brought about by the Suharto regime, campaigning on a platform for increased equity, leading to their election to power in 1999. They leveraged focus on social welfare and equity as a political tool to gain legitimacy amongst a public who were already protesting against Suharto's policies. After the electoral success of the party, the Megawati administration amended the Constitution in 2000 to include “the right to receive medical services,” highlighting the state's responsibility in ensuring health service provisions and sought to develop a social security system for all citizens (37, 38).

In Brazil, it was the process of democratization and the promulgation of the constitution, which led to the prioritization of healthcare and priority to the social sector more broadly. Brazil experienced a period of military dictatorship from 1964 to 1989. The last phase of the military dictatorship (1985–1990) marked the process of re-democratization in the country wherein a regime opposed to the dictatorship (Brazilian Democratic Movement Party) came into power (1986), a new constitution was promulgated (1988) and the popular presidential elections were carried out (1989) (39).

It was within this backdrop, with pressure from the Brazilian *sanitarista* (public health) movement (discussed in detail in the next section), that Brazil witnessed attention to the health sector. Amidst the economic crisis and democratization in the 1980's, the country witnessed the emergence of healthcare reforms which culminated in the recognition of health care as a right of citizenship and the creation of the public, universal Unified Health System (SUS) enshrined in the Constitution of 1988 (27). During the Constituent Assembly in the late 1980s, when Brazil was moving toward re-democratization, all the left oriented parties and the progressive segments of the major parties agreed upon the need for a public health system (40). Thus, in the new constitution, ‘it was the health sector that presented the most complete proposal both in terms of governing principles and in the organization of the system' (41).

#### Change in regime

The reforms in Turkey were situated in a political context of a change in political leadership, with the election of the AK Party in the early 2000's. The party came into being 15 months prior to coming into power in 2002, borne out of a separation from the major Political Islamist movement, presented as a “conservative democratic” party aiming to bring together various streams of centrist and rightist parties. In the 2002 general elections, the AK Party won by a majority and “ended a decade of poorly functioning coalition governments” (19). While the party adopted a neo-liberal approach to the economy, it had a clear preference to keep the role of the state intact on social and healthcare policy (4). The AK Party had seven main components in its party programme, one of which was dedicated to social policies, which included healthcare.

Consequently, when the AK Party came into power on a populist mandate, in order to appeal to its significant voter base of rural poor and urban slum dwellers, it focused on a pro-poor narrative. Yilmaz (4) finds that “Healthcare was key in the AKP's quest for power, and that the AKP used healthcare to influence people.” Yilmaz argues that the AK Party focused on social policies and healthcare reform specifically to distance itself from the Political Islamist movement it had emerged out of, as all parties affiliated to the movement had been shut down.

Health reforms, in the form of the Health Transformation Plan (HTP) implemented well, resulted in electoral success for the AK party over the years; in turn continuing the priority to healthcare, through which the party was able to leverage greater political legitimacy. Over a ten-year period, the percentage of the population satisfied with the healthcare system in Turkey increased from 39.5% in 2003 to 74.7% in 2013 (4, 19). Public opinion surveys indicated that the general public considered healthcare reforms as the party's most successful achievement (4).

Patton (42) argues that the party was able to push for reforms and deliver successfully on them due to a combination of factors including “a more stable government staffed by an AK Party majority in 2002, better fiscal management, and a demand for better healthcare from working class constituents in rapidly expanding urban areas.” The World Bank provided technical and financial assistance in introducing the reforms, with the relations between the Turkish government and the World Bank strengthened with the AK Party. Yilmaz (4) argues that the release of the World Bank's report on Turkey's healthcare system in 2003 was “influential in setting the main parameters of the political debates on Turkey's health- care system” and served as a reference point for the AK Party which was already motivated to reform the country's healthcare system.

Mexico witnessed the Vincente Fox led National Action Party (NAP) coming into power in the early 2000's after breaking the Institutional Revolutionary Party's (PRI) hold on presidential power for over 70 years. A new party and the political ideology of the health minister, Dr. Julio Frenk, when the party came into power, led to the initiation of health reforms in Mexico, largely termed as a minister driven reform (43). The minister had already led several academic efforts since the late 1980's to examine the challenges confronted by the Mexican health system and built on his expertise, as well as the support of the President and other stakeholders to drive the reform process from beginning to end, culminating in the creation of the System of Social Protection in Health (SSPH) and its health-care insurance component, the Seguro Popular (21).

Argentina witnessed one of the greatest economic and unemployment crises in the country's history in 2001, leading to disappointment with the political and economic situation in the country and citizens re-evaluating the presidency of Carlos Menem of the Peronist party in the 2003 general elections (44).

In the run up to the elections, Nestor Kirchner (who won the elections) ran on a center left platform and focused on production and work to battle the legacy of social exclusion bequeathed by the menemista mode' (44). Upon coming into power, President Néstor Kirchner, focused on the expansion of social rights for the country's population, including increased coverage of public health programs. The government of Argentina prioritized healthcare and invested in the health sector as part of its poverty alleviation programme (32). Various programs were introduced including Plan Nacer—a Maternal-Child Health Insurance program—aimed at increasing coverage of basic services among the uninsured population and improving the governance and efficiency of the health system (45, 46).

In Thailand, the push for reforms came from the newly elected political party, Thai Rak Thai,—a populist reform-oriented party led by Thaksin Shinawatra—who campaigned on a pro-poor agenda in the lead-up to the January 2001 elections (24). Thaksin Shinawatra, leader of the Thai Rak Thai party, taking note of the rural discontent against the incumbent of the pro-market, Democrat party, collaborated with a large and vocal civic group with rural roots (47) and promised universal health coverage in his campaign. This was subsequently implemented in the form of the 30 Baht Reform when the party came into power. Political actors and bureaucracy played an instrumental role in the introduction of universal health coverage in Thailand leading to its successful implementation in 2002 in the form of the “30 Baht” reforms.

Similar to Brazil, healthcare professionals came to occupy important positions in the government in Thailand, playing a significant role in pushing UHC on the agenda and ensuring its implementation. Two senior members of the party, including the future Deputy Prime Minister, were members of the Rural Doctor's Society (RDS)—a society formed in 1978 and instrumental in driving healthcare reforms in Thailand (explained in detail in the next session) and strong supporters of universal health coverage. Mor (48) argues that the victory of the TRT party and its pro poor agenda focusing on healthcare was also seen as a window of opportunity by members of the RDS who seized the opportunity and pushed for UHC in the country. United in their “deep core beliefs” around the importance of UHC, the doctors were crucial in driving the reform process. As senior members of the political party in power as well as in the Ministry of Public Health, they were able to bring healthcare to the political agenda. Kuhonta (24) argues that the new constitution introduced in 1997 significantly increased the power of the prime minister and allowed political dominance for Thaksin and TRT, with the context of the Asian financial crisis and the resulting economic hardships further helping to bolster the popularity of the party.

In the Philippines, the Benigno Aquino III presidency (2010–2016) followed the Gloria Macapagal Arroyo regime (2001–2010). The latter saw high inequalities in healthcare, reflected through high out-of-pocket health expenditures and impoverishment of poor households. Despite healthcare being free for poor households during the Arroyo regime, poor implementation led to inefficiency and corruption in the public healthcare systems (25, 26). The Benigno Aquino III presidency, consequently, sought to bring in radical change from his predecessor by focusing on effective implementation of social and economic welfare programs such as healthcare, education and employment. This focus was also motivated by his mother's [President Corazon Aquino, (1986–1992)] legacy and influence on his voter base (49). It was during the presidency of Corazon Aquino that healthcare saw an initial impetus, with the implementation of the Local Government Code (50), providing local government units the power to manage region specific health systems. This laid the foundation for the National Health Insurance Act (51), later establishing PhilHealth as a national health insurance body. This legacy played a key role in several health reforms undertaken by the Aquino III administration (52, 53).

The conditions during the Arroyo regime changed significantly with the Beningo Aquino III administration undertaking several health reforms to strengthen the roadmap toward Universal Health Care, and the commitment of the Aquino III Presidency was instrumental in establishing a strong social contract with the Filipino people (54, 55).

Healthcare prioritization in China saw a shift in the mid-1970's when it moved from a government led socialist economy to a market economy brought about by the privatization policies of the Deng Xiaoping administration (1978–1991). The shift to the market-based system started in 1978, when the policies of the centrally planned socialist system led to severe underemployment, low productivity, poverty, and famines. The shift to market economy was envisioned as a means to produce rapid economic growth, which also saw its effect on the health sector with greater push toward individual self-reliance. This change resulted in a significant reduction in government regulations within the healthcare sector which led to increased mark-up on drugs, and re-orientation of public hospitals into for-profit entities. These health practices led to health inequalities between rural and urban residents, poor quality of healthcare and increasing private health expenditure. The high healthcare costs and lack of insurance coverage prompted high public discontent and protests, picked up through media coverage (34).

At the same time, two other policy windows contributed to the change—the SARS outbreak and a transitional political leadership (34). The tumultuous time of the SARS outbreak and public unrest over high healthcare costs coincided with the national political transition (between November 2002 to March 2003) within the Chinese leadership which led to the start of the regime of President Hu and Premier Wen. The Hu-Wen administration had a different set of social values than their predecessors (Deng Xiaoping administration), and gave a higher priority to the health needs of Chinese rural and urban residents and considered a health safety net as crucial for people's wellbeing.

While healthcare is one of the core agendas of China's communist party ideology as part of its social welfare system, the healthcare reforms that took place in 2003 were influenced in large part by a need for the Chinese Communist Party to demonstrate good governance, as also a means to control the citizens' protests and reduce focus on the state's failure to provide access to quality healthcare. Thus, external pressures, citizen demand as well as the ill-effects of the SARS pandemic prompted the Hu-Wen government to provide basic insurance programs as well catalyzed the change for the 2009 reforms, which led to the establishment of universal healthcare for all Chinese residents (34, 56).

Our analysis of select countries suggests that while political transitions have been a common factor in shifting priority to social policy, it is not a necessary condition. The experience of Vietnam points to the initiation of healthcare reforms even in the absence of political transitions.

Vietnam, being a one-party led communist state, had an inherent mandate of providing access to healthcare to all its citizens as part of its socialist agenda (57), though, as pointed out earlier in this paper, 37% of the population lived under the $1.90 poverty line (2011 PPP), with high out-of-pocket expenditure on healthcare. In late 1980's, Vietnam was hit by a socio-economic crisis after the collapse of the Soviet Union which reduced foreign aid. This affected the government's ability to solely fund health care activities and ushered in a market economy policy with a socialist government structure. While this led to the privatization of healthcare, the government was careful to protect the interests of its people through a law on people's health protection (1989), and socio-economic development plans and budgets. The law signified the commitment of the Vietnamese Government to Universal Right to healthcare (58).

### Social context

The social context, in terms of citizen demand, social movements and the influence of policy actors constitutes the third pillar, which not only brings visibility to the issue but establishes it as a key national agenda. Agenda setting and political prioritization are influenced by various factors. Policy actors—including NGOs, civil society organizations, social workers and activists—can be key in influencing agenda setting and policy choice (10). Similarly, social movements can influence national agendas (13), as can demand from citizens for reforms. The presence of such social drivers, and their interaction with the political process, was visible in most of the countries studied, in their contribution to the creation or strengthening of incentives for political leaders in prioritizing healthcare.

Brazil saw policy actors playing a key role in the prioritization of healthcare reforms in the country. “Brazil's *sanitarista* (public health) movement had long advocated for more equitable health reforms and played a critical role in institutionalizing principles of universalism in the 1988 constitution, following the transition to democracy in 1985, and for the 1990 Unified Health System Law” (59). Various actors came together to give rise to a healthcare movement which sought to transform a segmented, fragmented, inefficient and exclusive healthcare system. These included academics working on preventive medicine or public health, administrators, and experts from the federal Ministry of Health and from the health bodies connected to the Ministry of Social Security, and other health professionals (27). They collaborated with social movements and progressive politicians to construct a reform agenda. In 1986 for example, at the Eighth National Health Conference, about 4,000 academics, administrators, health professionals, social movements, and ordinary citizens came together to advocate for the designation of health as a right. This led to the formation of the National Committee for Health Care Reform, which presented a proposal in front of the 1987–1988 National Constitutional Convention (27). The same health experts and members of the healthcare movement came to occupy important positions in the Ministry of Social Security and Assistance and the Ministry of Health which enabled them to push for healthcare reforms (60).

While health experts and social movements drove the healthcare agenda in Brazil, Turkey entered the new millennium with the population having increased expectations from the government including a demand for “decisive policies that would advance citizens” democratic rights; improve health and education services' (19). Citizen dissatisfaction with the socio-economic conditions of Turkey was visible in their discontent with the health system which came to light through the findings of a satisfaction survey by the Turkish Statistical Institute. The survey found 39.5% of the population being satisfied with health services in the country. This was lower than social insurance (40.2%), legal and judiciary (45.7%), and public security and order services (57.9%) (19).

China witnessed large citizens' protests following increasing health inequities due to the SARS outbreak and private healthcare costs. The citizen demand for healthcare was highlighted in 2005, when a national poll of over 3,000 people ranked healthcare systems as the topmost problem in China. International media picked up this issue and highlighted it, resulting in greater political focus (34).

In Indonesia, the introduction of the 2011 Badan Penyelenggara Jaminan Sosial (BPJS) law which mandated social security protection for all Indonesians, saw several organizations such as labor unions and NGOs coming together to form the Social Security Action Committee (KAJS) to ensure that the BPJS funds were directed into health insurance for all (35). Citizen protests in Indonesia and student movements played a critical role, with the student movement culminating in the formation of a political party.

Political attention to health in Thailand can be traced back to the times of King Rama VI (1910-25) which saw early investments in health system infrastructure (61). By the 1980's, a few policy elites in the Ministry of Public Health had started working on Universal Health Coverage. They included former student leaders who had fought against military rule in the 1970's and leaders of the Rural Doctor's Society (RDS); a society formed in 1978 and instrumental in driving healthcare reforms in Thailand. The RDS was formed by a group of doctors from elite medical universities in the country to support doctors working in rural areas and eventually became the institutional base for progressive reforms in the Thai health care sector (24). Over time, the doctors came to occupy important positions in the Ministry of Public Health, civil society and non-governmental organizations, and political parties, including in the Thai Rak Thai party which came into power in 2001.

## Discussion

An analysis of the nine countries reveals that healthcare reforms happened in the backdrop of an economic system marred by high rates of poverty and/or inequality, leading to high out of pocket expenditures on healthcare. At the time of reforms, most countries witnessed growth contraction, unemployment, inequality and citizen dissatisfaction with healthcare access, high rising costs of healthcare emerging from privatization of healthcare in some cases. Regional contexts were contributing factors: the Asian financial crisis for Thailand and Indonesia, the collapse of Soviet Union for Vietnam, and the transition from autocratic regimes in Brazil and Philippines.

Elections and the formation of a new government proved to be the catalyst and the momentum for reform in most countries. Turkey, Thailand, Mexico, Argentina, Philippines, China, Indonesia and Brazil, all witnessed the start of reforms when new governments came into power. Whether political transitions were a result of a democratization process or a change in leadership resulting from elections, our analysis found that healthcare reforms were invariably motivated by a need to gain political legitimacy by the incoming regime, or motivated by the political ideology of the new regime, or a combination of both.

Where a new regime was yet to establish its legitimacy with the voter base and form a social compact with citizens, the motivation was borne out of seeking political legitimacy through addressing a key and felt need amongst citizens; reaping electoral benefits from political capital formed. Seeking political legitimacy was a driving motivation for reforms in Turkey, Philippines, Indonesia, Brazil and China, all of which witnessed new political regimes in power. The A K Party in Turkey prioritised healthcare to differentiate itself from the previous Political Islamist movement and to gain credibility as a new party. Aquino III sought legitimacy when he came to power in Philippines, through countering the corruption ridden regime of his predecessor and simultaneously building on his mother's legacy (during whose regime healthcare received considerable attention) by focusing on social policy. The PDI-P party in Indonesia, that replaced the Suharto rule, sought political legitimacy through responding to citizen protests against Suharto and focused on social policy and equity, areas where the Suharto regime had failed. In the case of Argentina, Nestor focussed on a centre left campaign to distinguish himself from Menem and implemented social equity programs including healthcare reform upon coming into power. China, despite being an authoritarian regime, felt the need for political legitimacy for the new leadership, given large citizen protests against rising healthcare costs; leading to the prioritization of healthcare. Reforms in China were influenced by both: a need to seek political legitimacy, as also a response to an ideology of social welfare and equity.

On the other hand, certain new political regimes came into power with a foundational ideology of social welfare and equity, which formed the motivation and base for health reforms, as revealed by the experience of Brazil and Argentina. The motivation in Thailand was driven by a combination of the ideology of the network of bureaucrats who had for long engaged with healthcare, and the new political regime's need to seek political legitimacy through improved healthcare. In the case of Mexico, the ideology of the Health Minister played a key role in the prioritization of health. Vietnam was the outlier, where the introduction of reforms did not align with a new political regime. However, even then, it was the political ideology of social equity which was the driving force for the existing regime, leading to the prioritization of healthcare in the country.

The achievement of tangible improvement in benefits offered legitimacy to political regimes in two ways. One, it contributed to the legitimacy needed to sustain the government itself and second, it provided the legitimacy to undertake further reforms. This was then a reinforcing cycle, where key reforms, well implemented and effective in addressing critical needs, sustained governments, which in turn contributed to sustaining reforms. The experience of Turkey illustrates this well. Well implemented reforms fuelled expectations from citizens (at the very least, from those benefiting from the reforms), which led to increased citizen demand, creating the space for further reform.

While the countries examined in this paper point to ideology and/or the need to establish legitimacy as a driving force for attention to health and the initiation of reforms, the obvious question that emerges is what happens in contexts where neither of these can be a driving factor? It is possible that in some country contexts, neither political ideology nor political legitimacy is centred around issues of social equity. Would that then suggest the absence of political motivation for attention to social sectors in such cases? It does not have to, as our analysis of select countries reveals that the political motivation outlined above does not necessarily take root in itself. On the contrary, it is often driven by other factors and stakeholders such as varied policy entrepreneurs and advocates, who contribute to creating or strengthening incentives for political attention. The experience of some of the countries studied shows that internal and external advocacy played a key role in ensuring that key issues got highlighted and identified as those needing attention.

In the case of Brazil and Thailand, social movements and long-standing networks of doctors and public health professionals (*Sanitarista* movement and Rural Doctor's Society) played an important role in the prioritization of healthcare, by bringing visibility to a felt need and positioning health as a high-level priority. The voice of citizens and the role played by bureaucrats combined to create the context where health was viewed as a potential means to political legitimacy. In countries such as China, despite the ideological position on social welfare, citizen protests contributed significantly to the political regime viewing health as a pathway to strengthening legitimacy. Similar citizen protests were seen in Indonesia.

Research by Levitsky (62) outlines the importance of citizen awareness, voice and politicization in “removing the political cover for maintaining the status quo.” The role of social movements and other advocacy actors works to challenge a potential notion that given policies may be aligned with citizen choice or acceptable to citizens. The public questioning of such an assumption and expressed dissatisfaction with policies lifts the mask off what may be viewed as a minor problem and offers the platform to form a social contract with citizens through addressing the issue.

The role of citizens and social movements, and of policy entrepreneurs internal to the system, can be a key factor in influencing the motivation for political regimes, especially when such motivation is based on seeking political legitimacy. It is for citizens and movements to create the platforms that underline what would constitute, or contribute to, political legitimacy for a particular regime. Citizen voice by itself may not be enough, as evident from the experience of countries such as Brazil, which underline the need for clear pathways to and full proposals for reform, which need to complement citizen movements.

Based on the preceding analysis, we offer the following framework for attention to, and action on health.

What are the implications for India and similar countries, where progress toward universal health coverage has been slow. Analysing the experience of key health reforms in India shows that India is not an outlier to this framework. The National Rural Health Mission (NRHM) was introduced in 2005, soon after a new government, the Congress led UPA (United progressive Alliance) government came to power in 2004. The coalition government forged a Common Minimum Program, focusing on the needs of India's poor. The UPA government's focus on addressing not only basic unfulfilled needs of India's citizens but also their rights to human development, translated into a social equity oriented politics, in contrast to the prior regime's politics, which promoted an “India shining” narrative.

The National Rural Health Mission, a key reform in the health sector, was introduced soon after the formation of the UPA government, as did a health insurance scheme for the poor, Rashtriya Swasthya Bima Yojana (RSBY). These, combined with other social policy measures introduced during the UPA regime, such as employment, food and education guarantees, all of which were, could be seen to emerge at the confluence of the UPA's rights based ideology and their need for differentiating themselves from the previous regime and seeking political legitimacy through addressing structural needs; specially in the context of the criticism targeted at the previous ruling regime for ignoring social welfare and equity issues.

A second significant reform in India took the form of a tax funded health insurance for 40 percent of India's population, in the form of what is now called PM-JAY, introduced by the BJP government during its 2014–2019 term. While this could be viewed as a mere expansion of the previous insurance program (RSBY), the context for the new program was one where several states had already launched state specific insurance programs, and the national government did not have a health program that conveyed its commitment to social policy. It could be argued^2^ that the need to take a stewardship role, and be associated with a key health intervention that could counter the previous regime's NRHM, could be the possible driving force for the reform, which could provide potential benefits for the political brand of the new regime. The BJP regime that came to power in 2014 also sought legitimacy through welfare schemes^3^, though largely taking the shape of welfare handouts, which were quite distinct from the previous regime's focus on rights based entitlements. These strengthened their identity of being welfare oriented, contributing to their political legitimacy, and PM-JAY fit well into such a policy focus.

Importantly, neither of these reforms took place in themselves. Other stakeholders played a key role: civil society in the case of the UPA regime and bureaucrats in the BJP case. The need to build an identity distinct from the previous government, prompted UPA leaders to engage extensively with civil society leaders (through the National Advisory Council formed by Sonia Gandhi), which contributed to the setting of a social equity agenda^4^. UPA leaders were also influenced by global discourse on the macro economy and health^5^, which contributed to driving attention to health^6^. Bureaucrats, and institutions such as the NITI Aayog, similarly, played a key role in the launch of the PM JAY.

In conclusion, for contexts where neither political ideology nor the social contract with citizens centres on notions of social equity with health as a key element, a greater role from citizens and other actors, questioning the political legitimacy of the regime in power and highlighting the schisms in the social contract between the two may contribute to shifting the source of legitimacy for leaders. Voices, through electoral and other platforms, combined with clear pathways to addressing felt challenges, have a role to play in building a deeper social contract and accordingly shifting the incentives of political leaders.

## Data availability statement

The original contributions presented in the study are included in the article/supplementary material, further inquiries can be directed to the corresponding author/s.

## Author contributions

SV led the study, conceptualized the study, directed it, reviewed multiple versions, provided edits and changes, and write sections of it. SS and SM conducted desk research and prepared drafts. All authors contributed to the article and approved the submitted version.

## Conflict of interest

The authors declare that the research was conducted in the absence of any commercial or financial relationships that could be construed as a potential conflict of interest.

## Publisher's note

All claims expressed in this article are solely those of the authors and do not necessarily represent those of their affiliated organizations, or those of the publisher, the editors and the reviewers. Any product that may be evaluated in this article, or claim that may be made by its manufacturer, is not guaranteed or endorsed by the publisher.

## Footnotes

^1^List provided in an Appendix.

^2^Based in key informant interviews. List of stakeholders provided in Appendix.

^3^The provision of a cooking gas connection, agricultural cash transfers etc.

^4^Based on discussions with key stakeholders.

^5^WHO commission on Macro Economics and Health, 2001.

^6^Based on key informant interviews. List of stakeholders provided in Appendix.

## Appendix

List of stakeholders who provided input to this paper.

Hong Wang

Krishna Lavu

Louise Tillin

Magunta Reddy

Nachiket Mor

Nishant Jain

Owen Smith

Rajani Ved

Rifat Atun

Sanjay Kumar

Sasmit Patra

Sharbani Chakraborty

Siddharth Bhattacharya

Tania Dmytraczenko

Volkan Yilmaz.

Yamini Aiyer
